# Characterization of *Drosophila Saposin-related* mutants as a model for lysosomal sphingolipid storage diseases

**DOI:** 10.1242/dmm.027953

**Published:** 2017-06-01

**Authors:** Julia Sellin, Heike Schulze, Marie Paradis, Dominic Gosejacob, Cyrus Papan, Andrej Shevchenko, Olympia Ekaterina Psathaki, Achim Paululat, Melanie Thielisch, Konrad Sandhoff, Michael Hoch

**Affiliations:** 1 LIMES-Institute, Program Unit Development & Genetics, Laboratory for Molecular Developmental Biology, University of Bonn, Carl-Troll-Strasse 31, 53115 Bonn, Germany; 2 LIMES-Institute, Program Unit Membrane Biology & Lipid Biochemistry, c/o Kekulé Institute of Organic Chemistry and Biochemistry, University of Bonn, Gerhard-Domagk-Strasse 1, 53121 Bonn, Germany; 3 Max-Planck-Institute of Molecular Cell Biology and Genetics, Pfotenhauerstrasse 108, 01307 Dresden, Germany; 4 University of Osnabrück, Department of Zoology and Developmental Biology, Barbarastraße 11, 49076 Osnabrueck, Germany; 5 University of Osnabrück, Biology, EM unit, Barbarastraße 11, 49076 Osnabrueck, Germany

**Keywords:** Lysosomes, Sphingolipid degradation, *Drosophila*, Sphingolipidoses, Lysosomal storage diseases, Lipid profiling

## Abstract

Sphingolipidoses are inherited diseases belonging to the class of lysosomal storage diseases (LSDs), which are characterized by the accumulation of indigestible material in the lysosome caused by specific defects in the lysosomal degradation machinery. While some LSDs can be efficiently treated by enzyme replacement therapy (ERT), this is not possible if the nervous system is affected due to the presence of the blood-brain barrier. Sphingolipidoses in particular often present as severe, untreatable forms of LSDs with massive sphingolipid and membrane accumulation in lysosomes, neurodegeneration and very short life expectancy. The digestion of intralumenal membranes within lysosomes is facilitated by lysosomal sphingolipid activator proteins (saposins), which are cleaved from a prosaposin precursor. Prosaposin mutations cause some of the severest forms of sphingolipidoses, and are associated with perinatal lethality in mice, hampering studies on disease progression. We identify the *Drosophila* prosaposin orthologue Saposin-related (Sap-r) as a key regulator of lysosomal lipid homeostasis in the fly. Its mutation leads to a typical spingolipidosis phenotype with an enlarged endolysosomal compartment and sphingolipid accumulation as shown by mass spectrometry and thin layer chromatography. *Sap-r* mutants show reduced viability with ∼50% survival to adulthood, allowing us to study progressive neurodegeneration and analyze their lipid profile in young and aged flies. Additionally, we observe a defect in sterol homeostasis with local sterol depletion at the plasma membrane. Furthermore, we find that autophagy is increased, resulting in the accumulation of mitochondria in lysosomes, concomitant with increased oxidative stress. Together, we establish *Drosophila Sap-r* mutants as a lysosomal storage disease model suitable for studying the age-dependent progression of lysosomal dysfunction associated with lipid accumulation and the resulting pathological signaling events.

## Abstract (precis)

**Summary:** Detailed analysis of lipid accumulation, lysosomal dysfunction and neurodegeneration in a *Drosophila* sphingolipidosis model, suitable for studying the resulting pathological signaling events.

## INTRODUCTION

Lysosomes are membrane-bound organelles that have an acidic lumen, which is delimited by a single lipid bilayer membrane. A major role of the lysosome is the degradation and clearance of cellular waste as well as its recycling to feed into salvage pathways. Different routes are followed to transport extracellular and intracellular material into the lysosome for degradation. Extracellular material and integral membrane lipids and proteins reach the lysosome via specific endocytic mechanisms according to the type of the cargo (Conner and Schmid, 2003). Generally, intracellular materials are funneled into the lysosome through the process of autophagy, which is used by cells to capture their own cytoplasmic components, like macromolecules or whole organelles, destined for decomposition and recycling.

A major type of autophagy, called macroautophagy, starts with the biogenesis of autophagosomes, which form as double-membrane structures to sequester damaged, oxidized or dysfunctional intracellular components and organelles, and fuse with lysosomes for degradation (Feng et al., 2014). It is therefore often selective. For example, dysfunctional mitochondria are selectively removed in a process called mitophagy, which ensures mitochondrial quality control. Defects in mitophagy are associated with neurodegenerative diseases such as Parkinson's disease or Gaucher disease, underlining its importance for the organism, especially in the brain (Menzies et al., 2015; Osellame and Duchen, 2014).

Severe impairment of autophagic flux occurs in lysosomal storage diseases (LSDs), which are characterized by dysfunctional lysosomes accumulating non-degradable material (Lieberman et al., 2012). They are often caused by mutations in one of the many lysosomal hydrolases. Depending on the mutation, different molecule classes can be the primary storage material, like carbohydrates, proteins, or lipids. Some milder forms of LSDs are quite effectively treated by enzyme replacement therapies, which administer the missing enzyme into the blood stream (Pastores and Hughes, 2015). However, due to the existence of the blood-brain barrier, these therapies are not successful in more severe cases with neurodegenerative symptoms, which are frequently observed in LSDs with primary storage of sphingolipids, also called sphingolipidoses (Eckhardt, 2010).

Studies in mammalian cells have shown that sphingolipids are catabolized in the lysosomal compartment at the membrane-water interface in a stepwise fashion by soluble hydrolases with the help of lipid-binding and transfer proteins. These include the saposins (sphingolipid activator proteins) A, B, C and D, a group of four small proteins derived by enzymatic cleavage from a single precursor, prosaposin. Prosaposin is proteolytically processed in the lysosomal compartment to generate the four saposins, which present membrane bound sphingolipids to water-soluble exohydrolases for digestion. Whereas the inherited deficiency of a single saposin causes a late infantile lipid storage disease, the simultaneous loss of all four saposins in prosaposin deficiency causes ubiquitous storage of sphingolipids (like ceramide, glucosylceramide, lactosylceramide, sulfatide and gangliosides) in humans (Bradová et al., 1993; Burkhardt et al., 1997; Paton et al., 1992) and mice (Fujita et al., 1996) with very early lethality as a consequence. Most mice lacking prosaposin die neonatally due to an ichthyotic skin phenotype, or exhibit rapidly progressive neurological signs around day 20 and death by 35-38 days with massive sphingolipid storage comparable to the human disease (Doering et al., 1999; Fujita et al., 1996).

The pathological consequences of lysosomal storage and dysfunction are complex and still not clearly understood, since they involve many secondary effects – from specific defects like altered membrane composition (e.g. caused by sphingolipid shortage or accumulation) to impaired autophagy and other cell protective functions of lysosomes in general. The perinatal lethality caused by prosaposin deficiency hampers studies on the mechanisms of disease progression and age-dependent degeneration in the brain and other tissues of the body. Since the *Drosophila melanogaster* genome encodes a single prosaposin-like locus called *Saposin-related* (*Sap-r*)*,* we decided to study saposin dysfunction in this genetically tractable model organism.

## RESULTS

### *Sap-r* encodes the single *Drosophila* prosaposin orthologue

**Fig. 1.**
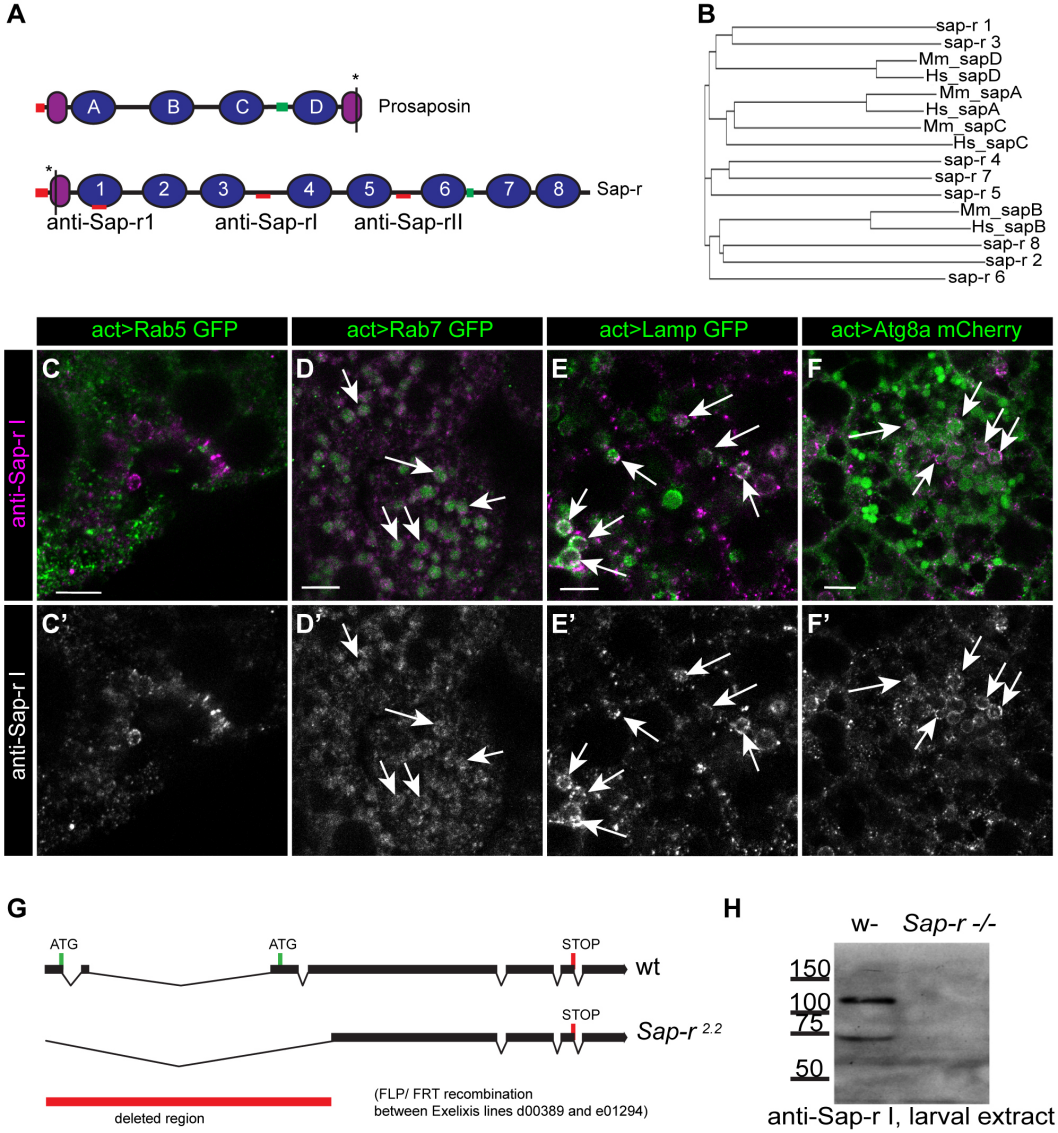
**Homology and subcellular localization of Sap-r protein and generation of a *Sap-r* mutant fly.** (A) Domain structure of *Drosophila* Sap-r and vertebrate prosaposin. Asterisks mark the presence of a conserved SORT-1 binding sequence in C-terminal prosaposin SapA and N-terminal Sap-r SapA domains. Position of epitopes for the three antibodies (anti-Sap-rI, -II and -1, respectively) used in this study are indicated as red bars. (B) ClustalW2 analysis of individual saposins from mouse, human and *Drosophila* shows that Sap-r 1 and 3 group with mammalian SapD, while Sap-r 2, 6 and 8 group with mammalian SapB. Sap-r 4, 5 and 7 cannot be grouped with any mammalian saposins. (C-F′) Antibody staining with anti-Sap-rI reveals that Sap-r is not localized to early endosomes (marked by Rab5-GFP, C), but can be found in late endosomes (Rab7-GFP-positive vesicles, D) as well as Lamp-GFP-positive lysosomes (E) and Atg8a-mCherry-positive autophagosomes (F) in the fat body of wild-type prepupae. Arrows indicate colocalization of the respective marker with anti-Sap-rI antibody staining. C′-F′ show the respective single channels of anti-Sap-r staining shown in C-F. (G) Genomic organization of the *Sap-r* locus. Start and Stop codons are marked as green and red lines, respectively. A mutant was generated by FLP-FRT recombination, deleting the region marked by a red bar, resulting in the allele *Sap-r*^2.2^. (H) Western Blot analysis of larval extracts, detected with the newly generated anti-Sap-rI antibody (epitope indicated in A), shows that *Sap-r^2.2^* is a null allele (also confirmed by real-time RT-PCR, data not shown). Scale bars: 10 µm.

*In silico* analysis indicates that the overall domain structure of Sap-r is very similar to the human prosaposin, containing so-called SapA domains, which are cleaved off in humans during processing, and SapB domains, which harbor the functional lipid binding domains. Whereas human prosaposin contains four SapB domains, which are processed to yield the saposins A, B, C and D, the *Drosophila* Sap-r protein harbors eight SapB domains, which we termed Saposin 1 (Sap 1) to Saposin 8 (Sap 8) (Fig. 1A). SapB domains are found in a number of lipid-binding proteins of the so called SAPLIP (Saposin-like proteins) family and are characterized by the presence of six cysteine residues that form three intramolecular disulfide bonds, and a number of conserved hydrophobic residues (Bruhn, 2005; Munford et al., 1995). All SapB domains, either of mammalian or *Drosophila* origin, show perfect conservation of these six critical cysteine residues responsible for formation of the stable saposin structure (Fig. S1A). ClustalW2 analysis of the SapB domains revealed that Sap 1 and Sap 3 are closest to mammalian SapD versions, and Sap 2, 6 and 8 group with mammalian SapB. Sap 4, 5 and 7 do not group with any of the mammalian counterparts (Fig. 1B). Both the mammalian and *Drosophila* proteins contain an N-terminal signal peptide for its targeting into the secretory pathway.

Throughout the animal kingdom, SapB domains are present in a number of proteins, which form the SAPLIP family of proteins, comprising about 235 members (Bruhn, 2005). A search for proteins with a similar domain structure as human prosaposin (isoform B) using the ‘domain composition’ algorithm of the SMART database (Letunic et al., 2015) revealed 145 hits, which we compared with respect to their species' phylogenetic position (Fig. S1B). The majority of 22 hits in the Arthropoda clade contained 8 SapB domains (13×8, 1×10, 1×9, 4×7, 2×6, 1×2). There were only two other entries containing more than 4 SapB domains (10 and 11 domains, respectively), which were predicted proteins from a sponge species. The sponges are a group of basal clades in the metazoan phylogenetic tree and do not belong to the Bilateria (Halanych, 2004). The only other non-bilaterian entries were proteins from Cnidaria species (which are sometimes grouped with the Bilateria into the Eumetazoa, to which the sponges do not belong), which contained 3 SapB domains. All hits in eumetazoan clades except for Arthropoda, a total of 121 proteins (including putative proteins), contained between 1 and 5 SapB domains, including the Cnidaria, which are an outgroup compared with vertebrates and arthropods, and the Platyhelminthes, which are more closely related to arthropods than vertebrates (Halanych, 2004). The even closer related Nematoda, which group together with arthropods in the clade of Ecdysozoa, contain a maximum of three SapB domains in the proteins found by a SMART domain selection search for SapB. It is therefore likely that the higher number of SapB domains are an autapomorphic feature of arthropods.

We analyzed the expression of *Sap-r* by *in situ* hybridization (Fig. S2) and found a strong ubiquitous expression pattern of *Sap-r* mRNA in early blastoderm embryos, suggesting a maternal contribution. In later stages, the highest expression can be observed in metabolic organs such as the gut, the fat body and the excretory Malpighian tubules, and in the central nervous system and in embryonic hemocytes. The elevated expression in these organs persists throughout larval development. In general, weak ubiquitous expression can be observed in all tissues, consistent with a function of Sap-r in all cells.

We raised antibodies against various Sap-r epitopes (indicated in Fig. 1A), all of which are specific and recognize the 106 kDa full-length Sap-r at >110 kDa (Fig. S3, Fig. 1H). The presence of smaller specific bands of ∼65 kDa and sometimes ∼35 kDa (Fig. S3) suggests that cleavage of Sap-r occurs, similar to the vertebrate prosaposin, which is cleaved by cathepsin D (CathD) in the lysosomes to yield the saposins A, B, C and D (Hiraiwa et al., 1997), although the exact recognition sites of CathD are not known. Mature vertebrate saposins have a size of only ∼12-16 kDa (Leonova et al., 1996), but we were not able to detect similarly small fragments in larval lysates. CathD seems to prefer hydrophobic regions as a cleavage site and cleaves with slight preference after a lysine residue (Sun et al., 2013), which makes *in silico* prediction of cleavage sites difficult due to the high number of hydrophobic and lysine residues in the saposins.

Consistent with the *Sap-r* mRNA expression pattern, we found ubiquitous expression of Sap-r protein throughout development with increased levels in metabolic organs such as the gut and fat body, and in the central nervous system where Sap-r is expressed in both glia and neurons (Fig. S4). Assuming that Sap-r is functionally conserved, it should localize like mammalian prosaposin to the endo-/lysosomal compartment. On the subcellular level, we indeed found Sap-r to be co-localized with Rab7-GFP, a marker for late endosomes (Fig. 1D, arrows), with Lamp1-GFP, which is present in lysosomes (Fig. 1E, arrows), and with Atg8a-mCherry in autophagosomes (Fig. 1F, arrows). We did not find Sap-r protein in early endosomes as marked by Rab5-GFP (Fig. 1C). Taken together, we can confirm that Sap-r localizes to the endo-/lysosomal compartment, suggesting a conserved function.

### *Sap-r* mutants are semi-lethal and show enlarged endo-/lysosomal compartments

**Fig. 2.**
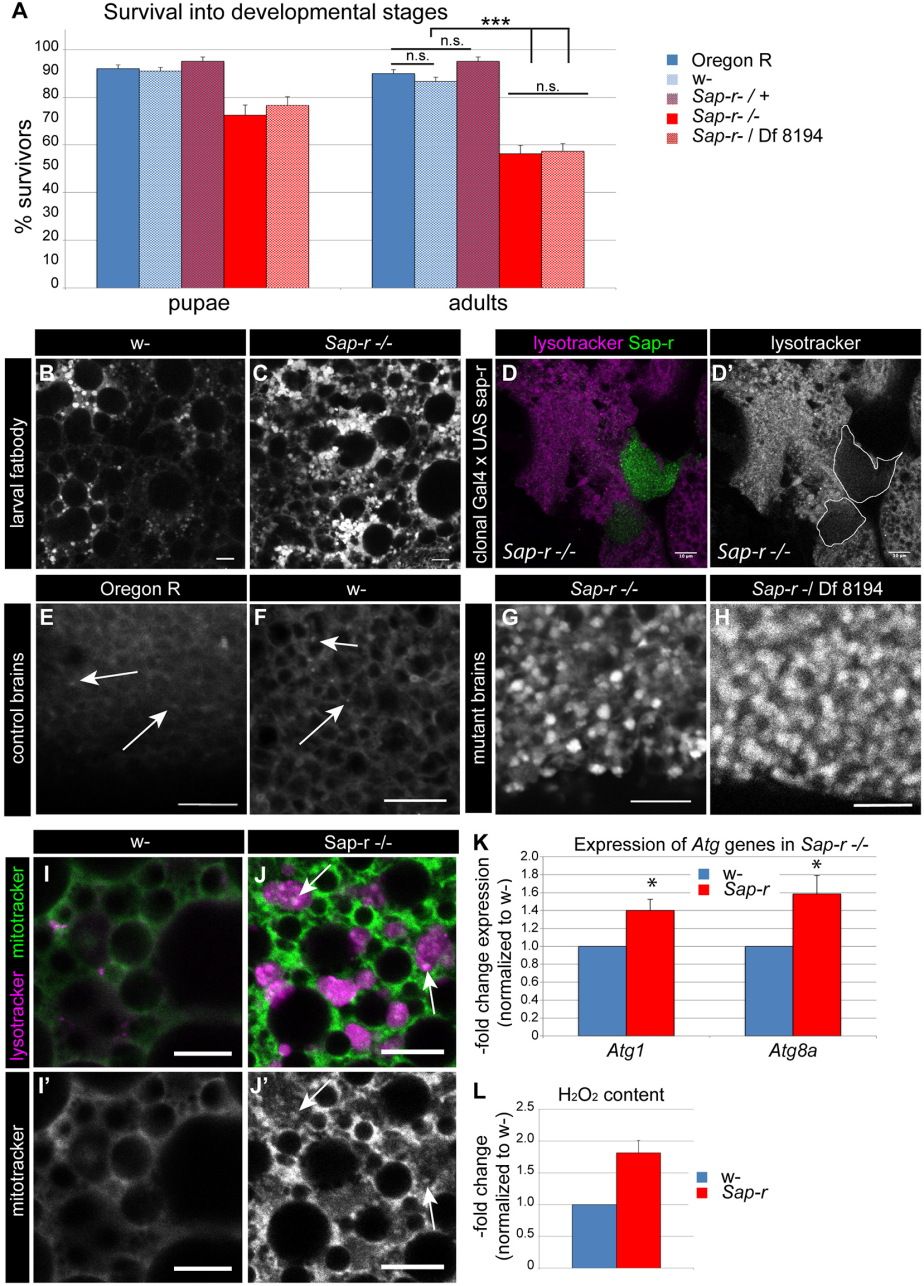
***Sap-r* null mutant flies show reduced viability, enlarged acidic compartments and increased oxidative stress.** (A) *Sap-r* mutant animals fed with apple juice agar/yeast paste mostly survive until pupation (∼70%, pupae), but only ∼50% survive metamorphosis (adults). Results are depicted as mean±s.e.m., *n*=6 (in groups of 20-25 individuals for each sample). (B,C) LysoTracker staining in larval fat bodies reveals increased size and number of acidic vesicles (endo-/lysosomal compartment) in *Sap-r* mutants compared with control larvae. (D) Reintroducing *Sap-r* function in clones in the mutant by use of the clonal driver act-FRT-CD2-FRT-Gal4 and a UAS-Sap-r line rescues the enlarged endo-/lysosomal compartment phenotype, showing a cell-autonomous function of Sap-r. (E-H) LysoTracker staining in adult brains reveals only a few, small lysosomes in either control (*w^1118^*; F) or wild-type (Oregon R; E) flies (arrows), but a massively enlarged acidic compartment in *Sap-r* homozygous (G) or transheterozygous (H) null mutants. (I,J) MitoTracker staining reveals mitochondria entrapped in autophagolysosomes (stained by LysoTracker) in *Sap-r* mutant fatbody cells (J), which is not observed in wild-type larvae (I), indicating increased mitophagy and/or reduced autophagic flux in the mutant. (I′,J′) Single green channels (MitoTracker staining) of I and J, respectively. Arrows indicate mitochondria entrapped in lysosomes. (K) *Atg1* and *Atg8a* expression as measured by real-time RT-PCR is increased in *Sap-r* mutant adult guts, indicating an increase in autophagy induction in *Sap-r* mutants, depicted as mean±s.e.m., *n*=5 (5-7 guts per sample). (L) H_2_O_2_ levels are increased in *Sap-r* mutant larvae, indicating increased oxidative stress. Values are mean±s.e.m., *n*=3 (8 larvae per sample). *P<0.05; ***P<0.001; n.s., not significant.

To analyze the function of *Sap-r*, we mutated the transcription unit by FLP-FRT based deletion, removing the first three exons of the gene, including the start codon and putative alternative start codon (Fig. 1G). The resulting *Sap-r^2.2^* mutant flies (referred to as *Sap-r* mutants from here on) are protein and mRNA null mutants as proven by western blot (Fig. 1H) and real time RT-PCR (data not shown), leading to a semi-lethal phenotype. On standard food (apple juice agar plates with yeast paste), only ∼55% of L1 larvae reach adulthood (Fig. 2A) and ∼70% reach the pupal stage. We also generated transheterozygous mutants over the *Sap-r*-uncovering deficiency Exel 8194 (*Sap-r^−^*/Df 8194) to diversify the genetic background of the mutants, which survive at the same rate as the homozygous *Sap-r* mutants. As control flies, we used *w^1118^*, which were used for generation of the parent lines of the mutant and therefore match the genetic background the best. They survive to ∼90% into adulthood, similar to wild-type Oregon R flies, as well as flies heterozygous for the *Sap-r* mutation (*Sap-r^−/+^*) (Fig. 2A). Slightly lower survival rates of the *Sap-r* mutants were observed on standard cornmeal food (Fig. S5), which is richer in carbohydrates, but contains less yeast-derived protein.

Since a reduced or abolished ability to degrade sphingolipids is expected to lead to a severe lysosomal storage defect, we used LysoTracker Red dye to stain the acidic compartments (late endosomes, lysosomes and autophagososmes) of control and *Sap-r* mutant flies in larval (Fig. 2B-D) and adult stages (Fig. 2E-H). We found that acidic vesicles in the mutants are dramatically increased in size and number compared with control or wild-type animals (*w^1118^* and Oregon R, respectively). To minimize the influence of genetic background, we also analyzed animals transheterozygous for *Sap-r* over Df 8194 with similar results (Fig. 2H). The severe lysosomal storage phenotype is clearly visible in almost all organs at larval stages. Among the organs most severely affected in larvae are the fat body (Fig. 2B,C), the tracheae (Fig. S6A,B) and the brain (Fig. S6C). The storage phenotype gets more pronounced with age (compare Fig. S6C with Fig. 2G,H for larval versus adult brain staining). To prove that lysosomal compartment enlargement is a direct consequence of loss of *Sap-r* function, we reintroduced Sap-r by using the act>CD2>Gal4 clonal driver line and a UAS-Sap-r construct. Indeed, in clones expressing UAS-Sap-r, LysoTracker Red staining is drastically diminished compared with the surrounding *Sap-r^−/−^* cells (Fig. 2D), demonstrating a cell-autonomous function of Sap-r in normal lysosomal function. Ubiquitous overexpression of Sap-r in either wild-type or *Sap-r* mutant background, however, leads to complete embryonic lethality, indicating that the correct dosage of Sap-r expression is important for embryonic development.

Lysosomal dysfunction caused by the inability to degrade sphingolipids consequentially leads to a block in autophagic flux. Dysfunctional mitochondria, delivered to the lysosome via autophagy, cannot be degraded and accumulate in autophagolysosomes. Consequently, increased oxidative stress and defective mitochondria quality control is associated with LSD pathologies in mammals and contributes to neurodegeneration (Osellame and Duchen, 2014). In our *Sap-r* mutant, we were indeed able to observe mitochondria (stained by MitoTracker Green) inside acidic vacuoles (stained by LysoTracker Red) in mutant larval fat body cells (Fig. 2J). In contrast, mitochondria in autophagolysosomes cannot be observed in control animals (Fig. 2I). This can be interpreted as an arrest in autophagic flux in *Sap-r* mutants (although the formation of autophagosomes, their acidification and the targeting of mitochondria for autophagy in general obviously is unaffected). Additionally, it could be caused by an increase in mitophagy induction. Accumulation of defective mitochondria will probably lead to increased oxidative stress. Indeed, our *Sap-r* mutants show strongly elevated H_2_O_2_ levels (Fig. 2L), indicating increased oxidative stress.

While lysosomal dysfunction ultimately leads to the inability to recycle autophagic content and impaired autophagic flux, a typical feature of many LSDs is also an increase of autophagy induction (Lieberman et al., 2012), both of which contribute to the enlarged acidic compartment in LSDs. To analyze if the enlarged acidic compartment in *Sap-r* mutants is not only due to impaired autophagic flux, as expected in the absence of sphingolipid activator proteins, but also due to increased autophagic induction, we analyzed the expression of *Atg1* and *Atg8a* (homologue of human LC3) by real-time RT-PCR, both of which code for proteins involved in autophagy initiation and which are transcriptionally upregulated when autophagy induction increases (He and Klionsky, 2009). Their expression is indeed significantly elevated in larval and adult stages in *Sap-r* mutants compared with that in control flies (Fig. 2K), indicating that increased autophagy induction also contributes to the enlarged acidic compartment in *Sap-r* flies. Considering that increased induction of autophagy is futile due to the block in autophagic flux, this will likely contribute to further accumulation of defective mitochondria in autophagolysosomes. Oxidative stress would thereby further increase, which could ultimately increase neurodegeneration and overall lethality.

**Fig. 3.**
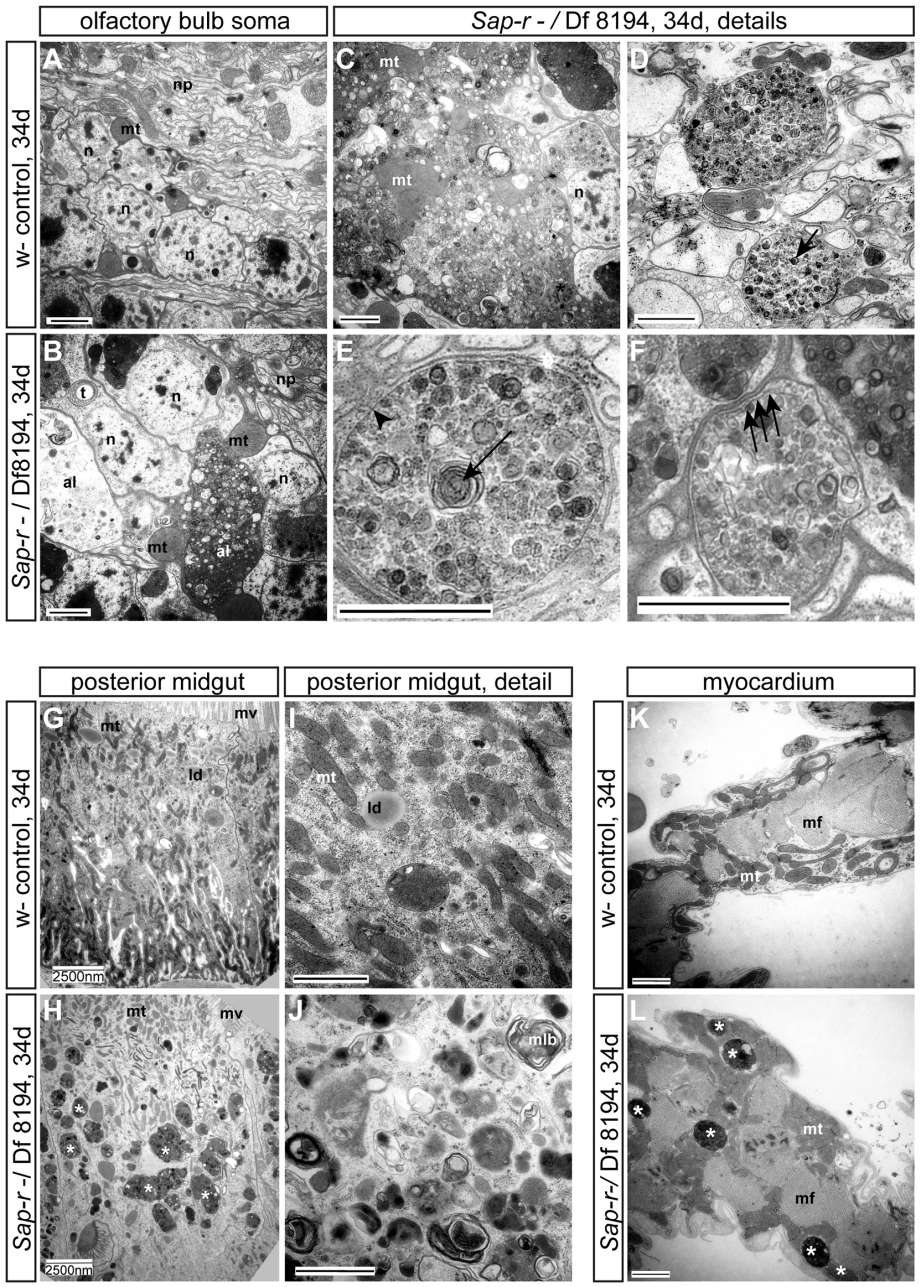
**Ultrastructural analysis reveals massive lysosomal storage and dysfunction in aged *Sap-r* null mutant flies.** A, G, I and K show control animals, all other images represent *Sap-r*^−^/Df 8194 mutants. (A,B) The soma surrounding the olfactory bulb contains massively enlarged late autophagolysosomes (al) with multivesicular and multilamellar structures in *Sap-r* mutants (B) compared with the wild type (A). The somata appear swollen and distorted. (C) In extreme cases, gigantic organelles containing stored material fill the majority of the soma in *Sap-r* mutants. (D,E) Autophagolysosomes containing multilamellar structures (arrows) are found throughout the brain of *Sap-r* mutants. Shown are examples from neuropile regions. (F) As a comparison, an autophagosome containing sequestered cellular material is depicted, recognizable by the double delimitating membrane (arrows). Autophagolysosomes are delimited by a single membrane (compare with E, arrowhead). (G-J) Massive lysosomal storage was observed in the posterior midgut of *Sap-r* mutants (asterisks in H). Numerous multilamellar bodies were observed (J, mlb). (K,L) The adult myocardium of *Sap-r* mutants contains huge autophagolysosomes (L, asterisks), which were never seen in control specimens (K). al, autophagolysosome; ld, lipid droplet; mlb, multivesicular body; mf, muscle filament; mt, mitochondrium; mv, microvilli; n, nucleus; np, neuropile; t, trachea. Scale bars: 1000 nm (A-F,I-L) and 2500 nm (G,H).

To evaluate the lysosomal storage phenotype on the ultrastructural level, we analyzed aged animals transheterozygous for *Sap-r* over Df 8194 by transmission electron microscopy (TEM). We found numerous enlarged autophagosomes, autophagolysosomes and multilamellar structures in all areas of the brain. The most severe lysosomal storage phenotype was observed in the soma-containing areas (Fig. 3A,B, soma surrounding the olfactory bulb), where sometimes gigantic autophagic structures with a diameter of up to 6 µm were found (Fig. 3C; as comparison, nuclei in that area have a diameter of ∼3 µm). However, enlarged multivesicular and multilamellar bodies were found in all areas analyzed, including in neuropile regions (Fig. 3D,E). Autophagosomes sequester materials destined for degradation and can be recognized by their double delimitating membrane (Fig. 3F, arrows). After fusion with lysosomes to yield degradation-competent autophagolysosomes, only a single delimitating membrane can be seen (Fig. 3E, arrowhead; for an overview on autophagic and lysosomal ultrastructure in neurons, see Nixon, 2007). Indeed, TEM analysis confirmed the presence of undegraded autophagocytosed material inside autophagolysosomes, including mitochondria (Fig. 3C), confirming a block in autophagic flux in *Sap-r* mutants. Furthermore, we also found massive levels of lysosomal storage in the posterior midgut (Fig. 3G-J, asterisks in H) and the adult heart (Fig. 3K,L), where huge autophagolysosomes were observed between the myocard-surrounding mitochondria (asterisks in Fig. 3L).

### *Sap-r* mutants show progressive neurodegeneration and motor function decline

**Fig. 4.**
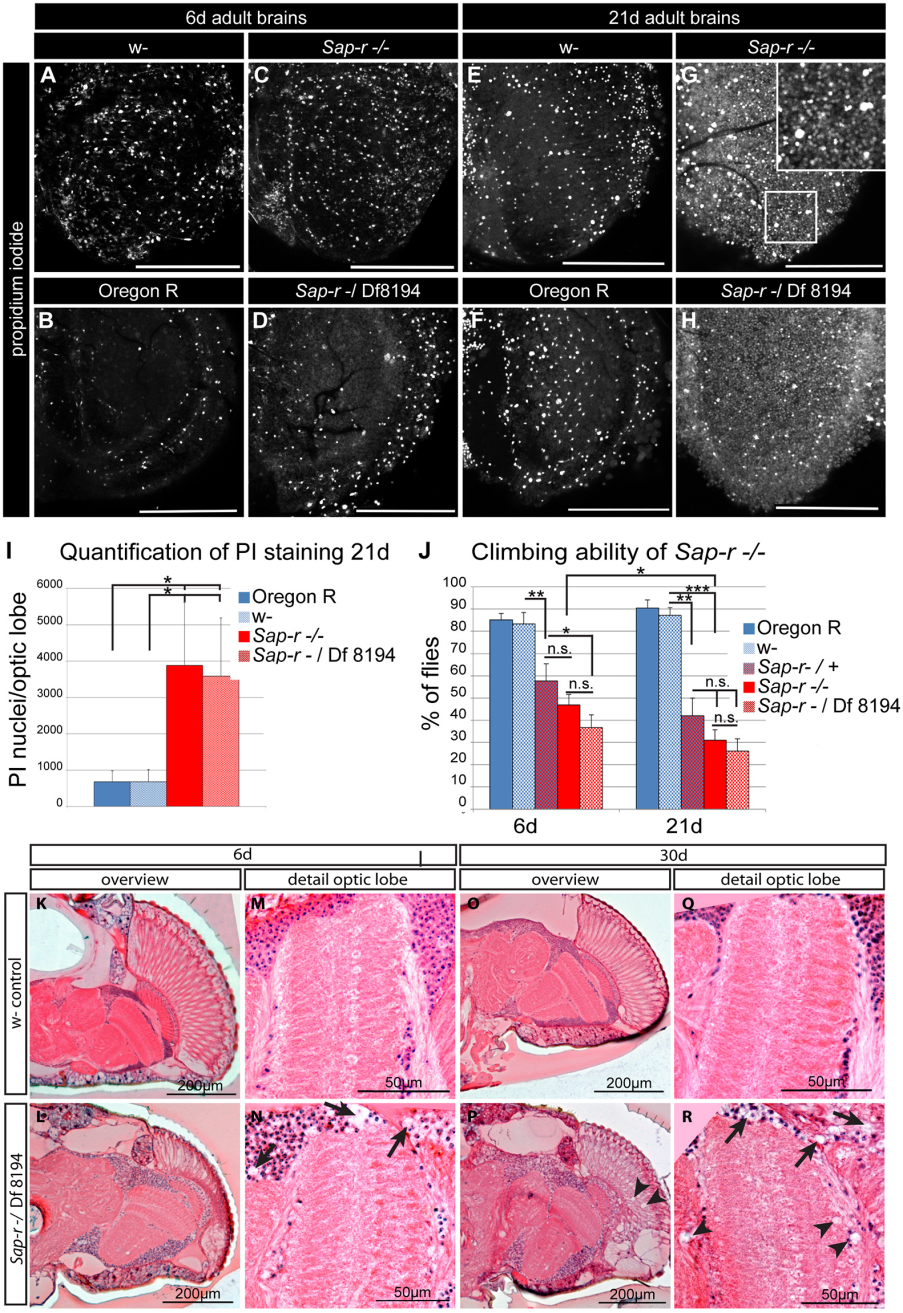
**Progressive neurodegeneration occurs in *Sap-r*-deficient flies.** (A-H) Propidium Iodide (PI) staining of adult brains after 6 days (A-D) and 21 days (E-H) reveals an increased amount of dead (i.e. PI positive) cells after 21 days in *Sap-r^−/−^* and *Sap-r^−^/*Df 8194 null mutants, indicating progressive neurodegeneration. Scale bars: 100 µm. (I) Quantification of PI-positive nuclei in 21 day optic lobes. Depicted are mean±s.e.m., *n*=5. *P<0.05; **P<0.01;***P<0.001; n.s., not significant. (J) Negative geotaxis assay to observe motor function decline. Climbing ability in 6-day-old mutant flies is reduced by ∼50% compared with the wild type, while after 21 days, the difference between wild-type and mutant flies is increased to ∼70%. Values are mean±s.e.m., *n*=5. Climbing goal was 8 cm in 20 s. (K-R) Hematoxylin and Eosin staining of brain sections. In 6-day-old *Sap-r^−^/*Df 8194 null mutants, neuronal somata appear swollen and spongy, with obvious holes, which is still present at 30 days (arrows in N and R). At 30 days, small lesions appear in the retina (arrowheads in P) and the neuropile regions (arrowheads in R).

A typical feature of impaired lysosomal sphingolipid degradation is neurodegeneration. We therefore stained brains of young and aged adults with propidium iodide (PI) to reveal dead cells in control animals (*w^1118^*, as this is closest to the genetic background of our mutant), wild-type Oregon R flies, *Sap-r* homozygous mutants, and, to provide a more diverse genetic background, transheterozygous *Sap*-*r^−^*/Df 8194 mutants. While we did not observe any obvious differences in 6-day-old brains, we did find an increase of dead cells in *Sap-r-*deficient brains of either genotype at 21 days (Fig. 4G,H, compare with E,F). Many small PI-positive nuclei appear in aged mutants (see Fig. 4G, inset, for higher magnification). Increased amounts of dead cells in *Sap-r^−/−^* or *Sap-r^−^*/Df 8194 mutants were confirmed by quantification using ImageJ (Fig. 4I). Quantification also showed that the absence of the *white* gene function has no influence on the number of dead cells found in the brain, as Oregon R wild-type flies show the same number of PI-positive nuclei as *w^1118^*, which is important considering that our mutant flies also lack the *white* gene function as they originate from a *w^1118^* parent stock.

Next, we tested homozygous *Sap-r* mutant adults, as well as transheterozygous mutants over Df 8194 to diversify the genetic background, for proper motor function and performed climbing assays. *Drosophila* shows negative geotaxis climbing behavior, which is tested by measuring the ability of flies to climb a certain distance in a given time. Decline in climbing ability correlates with neurodegeneration and indicates motor function decline (Pendleton et al., 2002).

While young adults (6 days after hatching) showed obvious defects in negative geotaxis behavior, the performance of 21-day-old adults further dropped significantly to ∼30% success, clearly indicating a progressive motor function decline in aging *Sap-r* mutant flies (Fig. 4J). Surprisingly, animals heterozygous for *Sap-r* showed also a significant reduction in climbing ability as compared with *w^1118^* control animals, suggesting that a reduction of *Sap-r* gene dosage already leads to neurodegenerative effects. Since *Sap-r^−/+^* animals survive into adulthood similar to wild-type flies (Fig. 2A), this dosage reduction seems to be less important in earlier stages of development.

**Fig. 5.**
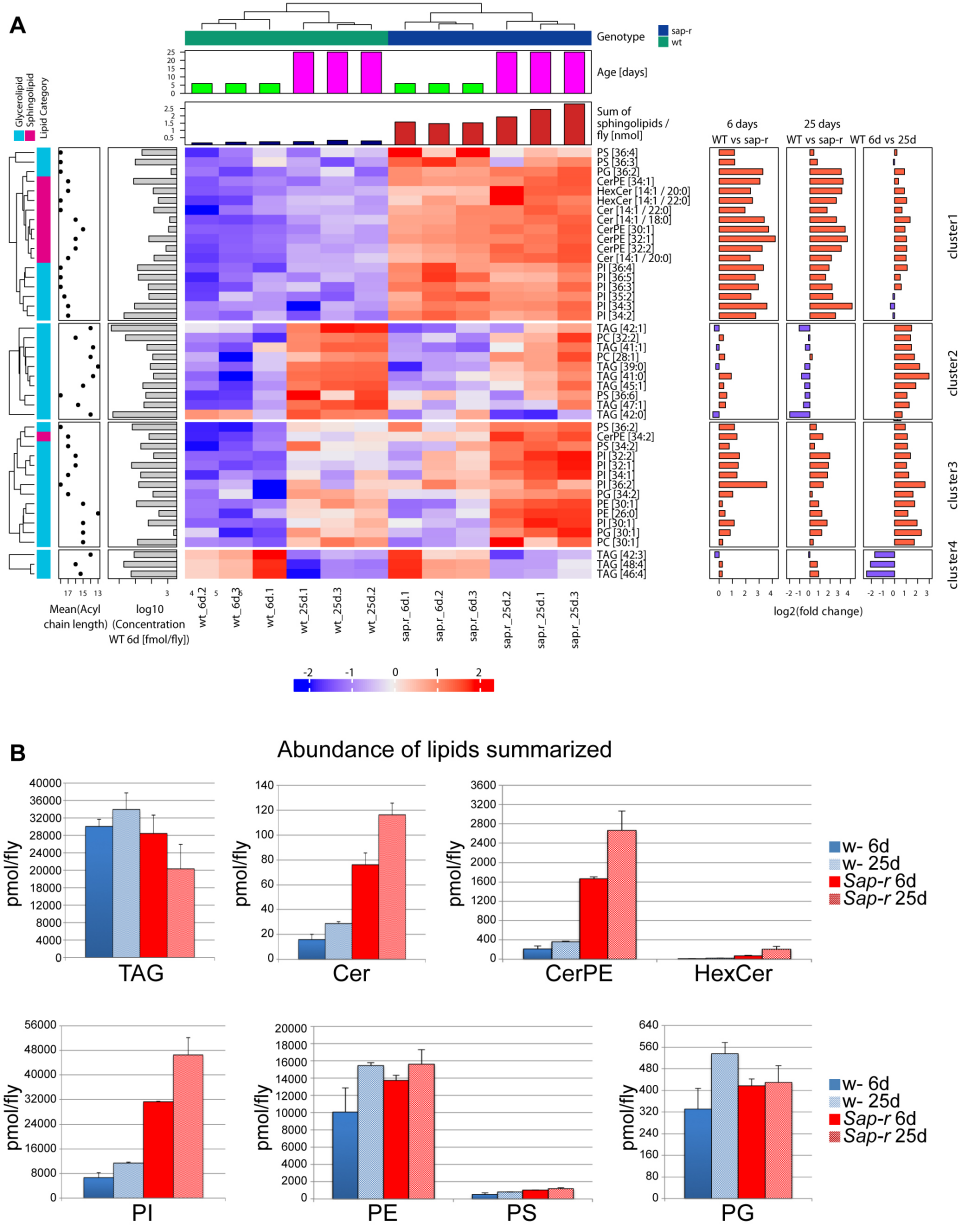
**Summary of MS lipid profiling: heatmap and quantification.** (A) Results from MS lipid profiling were clustered according to Materials and Methods, resulting in four main clusters. Cluster 1 comprises lipids enriched in *Sap-r* mutants, more or less independent of the age of the flies (only minor changes between wild-type (WT) 6- and 25-day-old flies or *Sap-r* 6- and 25-day-old flies). The lipids in the second cluster accumulate with age in both wild-type and *Sap-r* mutant flies, but to a lesser exent in *Sap-r* mutant flies (see summary on right hand side, 25 days, control versus *Sap-r*: negative log2 fold change). Cluster 3 comprises lipids that are more abundant in *Sap-r* mutants (young and old flies) and accumulate in both genotypes to a similar extent with age. Cluster 4 comprises lipids that are depleted with age (negative log2 fold change in wild type at 6 days vs 25 days), and behave similarly in *Sap-r* and wild-type flies. The majority of sphingolipids fall into cluster 1 (accumulation in *Sap-r* of any age, no accumulation in wild type), suggesting a function of *Sap-r* in spingolipid degradation homologous to vertebrate prosaposin (see also summary on top of the heat map: sum of sphingolipids/fly in nmol is increased in *Sap-r* at both 6 days and 25 days of age). Chemical structural information (lipid class, mean acyl chain length) and absolute abundance of individual lipids from young wild-type flies are annotated on the left of the heatmap and fold change values for genotype and aging comparisons on the right. (B) Summarized abundance of several lipid classes measured by MS are depicted as mean±s.d. (*n*=3) in pmol/fly.

To further analyze neurodegeneration in the absence of *Sap-r* function, we analyzed the brain of *Sap-r^−^/*Df 8194 mutants and *w^1118^* control animals at 6 days or 30 days of age by Hematoxylin and Eosin staining of histological sections (Fig. 4K-R). While overall morphology of *Sap-r* mutant brains seems normal, the Hematoxylin-positive neuronal somata appeared swollen and spongy at both time points, with obvious holes, possibly resulting from stored lipids that were washed away during the fixation process (Fig. 4N,R, arrowheads), consistent with the results from TEM analysis (Fig. 3), which revealed the most severe storage phenotype in soma regions. In older animals, neurodegeneration is also apparent in the brain neuropile in the form of small lesions (Fig. 4R, arrows). However, these lesions are relatively mild compared with some other neurodegenerative fly models, such as Swiss cheese (Kretzschmar et al., 1997), even though the number of PI-positive dead cells in aging brains is massively increased in the mutant (Fig. 5I), possibly hinting at problems with the removal of dead cells as a consequence of lysosomal dysfunction.

### Lipid profiling reveals sphingolipid storage in *Sap-r* mutants

**Fig. 6.**
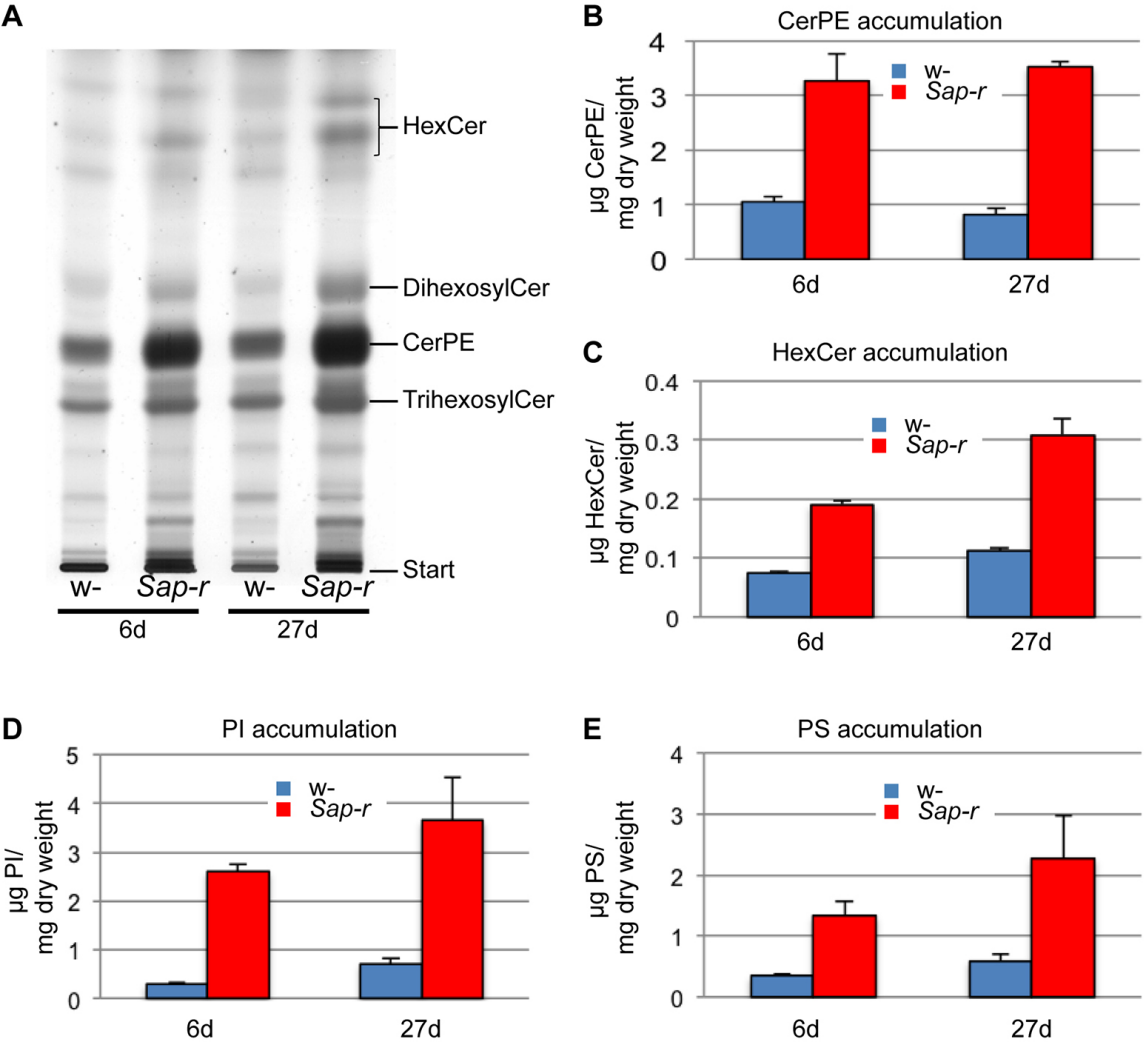
**Sphingolipids and phospholipids accumulate in adult Sap-r-deficient *Drosophila.*** (A) TLC analysis of neutral sphingolipids. Neutral lipids obtained after alkaline hydrolysis (from 10 mg *Drosophila* homogenate dry weight) of adult (6 days) and aged (27 days) control (w-) and *Sap-r-*deficient (Sap-r) *Drosophila* were applied to silica TLC plates that were developed with chloroform/methanol/water (60/25/4, v/v/v) for glycosylated ceramides and ceramide phosphoethanolamine (CerPE) before staining with copper (II) sulfate. CerPE and hexosyl ceramide (HexCer), as well as di- and trihexosyl ceramide are increased in the knockout. The HexCer double band reflects heterogeneity of its ceramide anchor composition. (B-E) Densitometric evaluation of CerPE (B), HexCer (C) and of the phospholipids PI (D) and PS (E) after TLC lipid analysis. Results are given in µg lipid/mg (*Drosophila* homogenate) dry weight. Values are mean±s.e.m. (*n*=4, PI and PS; *n*=6, CerPE and HexCer).

In order to investigate the lysosomal storage material in *Sap-r Drosophila* mutants, we analyzed the lipid composition of young adult (6-day-old) and aged (25- to 27-day-old) control and *Sap-r* mutant flies using mass spectrometry (MS, Fig. 5) and thin layer chromatography (TLC, Fig. 6) analysis. For MS analysis, we used 5 *Drosophila* as described in the material and methods section. For TLC, we used a larger sample of up to 150 flies. Results were consistent using both methods, although there were some minor differences in the extent of the storage.

Sap-r-deficient *Drosophila* exhibit a more than 3-fold increase of ceramides (Cer) in mass spectrometric analysis compared with levels in the control (Fig. 5B). The main ceramide species are [14:1/18:0], [14:1/20:0], [14:1/22:0] (Fig. 5A) and [16:1/22:0] (data not shown). Hexosylceramide (HexCer) levels are increased almost 6-fold in younger flies and almost 10-fold in 25-day-old flies (Fig. 5B), the main HexCer species being [14:1/20:0] (Fig. 5A). TLC analysis revealed a more than 3-fold increase of ceramide-containing neutral glycosphingolipids, i.e. mono-, di- and trihexosyl-ceramides (Fig. 6A,C, HexCer, DihexosylCer and TrihexosylCer).

The accumulation of ceramides and glycoceramides corresponds to the findings in human prosaposin-deficient patients and the homologous mouse model (Bradová et al., 1993; Fujita et al., 1996).

Phospholipids, such as phosphatidylinositol (PI) and, to a minor extent, phosphatidylserine (PS), are increased in Sap-r deficient *Drosophila* (Fig. 5A,B and Fig. 6D,E). PI main species [20:2/16:1] and [18:2/16:0] are increased more than 4-fold (Fig. 5A). Alterations in the phospholipid content have not been reported for prosaposin-deficient human tissues (Bradová et al., 1993).

In contrast to the situation in prosaposin-deficient mammals, ceramide phosphoethanolamine (CerPE) is one of the main storage substances in Sap-r-deficient *Drosophila.* In mass spectrometric analysis, its main species [32:1], [32:2], [34:1], and [34:2] are elevated almost 6-fold in young and 7-fold in aged Sap-r deficient *Drosophila* in comparison to control (Fig. 5A) and roughly 3-fold in TLC (Fig. 6B). CerPE is a minor species in mammalian tissues and is not accumulating in Prosaposin deficiency. In insects it takes over similar functions as mammalian sphingomyelin, which is not severely affected by the absence of Prosaposin in mice or human patients, probably since its catabolic enzyme acid sphingomyelinase contains a saposin-like domain (Kölzer et al., 2004).

In contrast to the other major lipids discussed so far, the total content of triacylglycerols (TAGs) is significantly lower in *Sap-r*-deficient flies compared with control flies (Fig. 5B). Decreased TAG levels in mutant flies possibly hint at changes in lipid metabolism or increased energy demands in the absence of Sap-r function. However, TAGs with longer acyl chains drop with age in both the mutant and control, while some shorter-chain TAGs accumulate in control files, but drop in *Sap-r* mutants (Fig. 5A).

### *Sap-r* mutants show altered sterol distribution

**Fig. 7.**
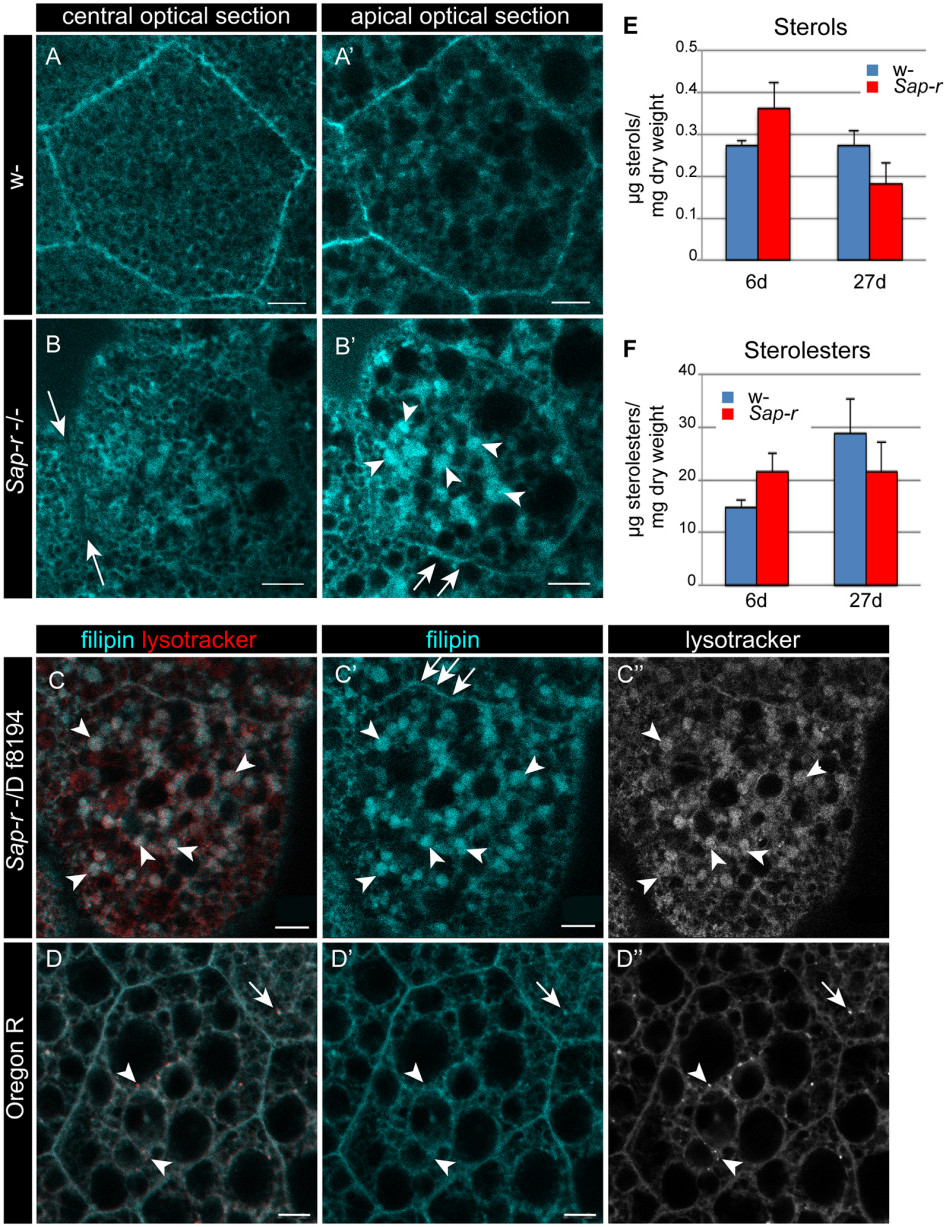
**Local sterol depletion at the plasma membrane and sterol accumulation in lysosomes.** (A,B) Larval fat bodies stained with filipin to reveal sterols. Mutant fat body cells have decreased sterol levels at the basolateral plasma membrane (arrows) concomitant with sterol accumulation inside the cell (B′, arrowheads). A and A′ as well as B and B′ show two different optical sections of the same cell. (C-D″) Double staining with LysoTracker Red and filipin shows that sterol accumulation occurs in the acidic compartment of *Sap-r* null mutants. Filipin accumulation occurs similarly in *Sap-r^−/−^* and *Sap-r ^−^*/Df 8194 mutants (compare C′ with B′). In wild-type larval fatbodies (D), some small LysoTracker-positive vesicles occur, which sometimes also are stained by filipin (arrow), but sometimes are not (arrowheads). (E,F) Densitometric analysis of TLCs of sterols (E) and sterolesters (F) reveals that overall sterol and sterolester content in adult *Sap-r* mutants do not increase after 6 days or 27 days of life. Values are mean± s.e.m. (*n*=4 for sterols and *n*=6 for sterolesters).

Filipin staining of larval fatbody tissue indicates that the sterol distribution is altered in *Sap-r* mutants, probably as a secondary effect due to close interaction of sphingolipids and sterols in membranes. In control fatbody cells, filipin staining predominantly marks the basolateral plasma membranes, where the main fraction of sterols (in *Drosophila* mainly ergosterol, zymosterol, campesterol, brassicasterol, and others, depending on nutritional availability, Carvalho et al., 2012) is localized (Fig. 7A,A′). In *Sap-r* mutant cells, filipin staining is strongest in vesicles inside the cell (Fig. 7B′, arrowheads) and the plasma membrane staining is severely reduced (Fig. 7B,B′, arrows), which is also observed in *Sap-r^−^*/Df 8194 larvae (Fig. 7C,C′). Double staining with Filipin and LysoTracker confirms that sterols accumulate in the acidic compartment of the cells (arrowheads in Fig. 7C,C′), while wild-type fatbody cells display some lysosomes devoid of Filipin staining (Fig. 7D, arrowheads). Total sterol and sterolester content, however, is more or less comparable in *Sap-r* and control adults of 6 or 25 days of age (Fig. 7C,D), indicating that the observed staining differences result from sterol misdistribution, not accumulation.

## DISCUSSION

In recent years, *Drosophila* has gained traction as an efficient model to study lipid metabolism in general (for a review, see Kühnlein, 2012) and sphingolipids in particular (Acharya and Acharya, 2005; Kraut, 2011; Bauer et al., 2009; Voelzmann et al., 2016). However, there is still a lack of knowledge concerning the degradation of membranes and in particular sphingolipids in *Drosophila*. In the present study we identify Sap-r as a novel regulator of sphingolipid degradation and lysosomal function in *Drosophila*. Our work characterizes the consequences of blocked sphingolipid degradation in the fly and uncovers the functional conservation of *Drosophila* Sap-r with the human prosaposin. MS lipid profiling revealed that all major sphingolipid classes accumulate with age in the null mutant, concomitant with enlarged acidic compartments, reduced viability and neurodegeneration. Interestingly, we also observe accumulation of a number of phospholipid species, in particular PI, which is noteworthy because its phosphorylated forms, the phosphoinositides, are involved in many signaling pathways, among them the induction of autophagy by PI3-kinase class 3/Vps34 in a complex with Beclin-1 (Atg6 in *Drosophila*) (Jaber and Zong, 2013). It is, however, unclear if they are altered due to differences in signaling, caused by secondary effects of altered membrane composition and other downward pathological consequences, or if they accumulate in lysosomes as secondary storage material, when overall lysosomal function declines as a result of increased storage, thereby possibly influencing downward signaling events directly.

Furthermore, we find that sterol distribution, but not overall content, is altered in our fly model, leading to local sterol depletion at the plasma membrane. This could lead to differences in many signaling pathways dependent on the correct lipid composition of membranes, as was shown, for example, for EGF signaling (Coskun et al., 2011). Our fly model therefore can be used to further unravel the downstream effects caused by altered signaling, which might be relevant in the context of human lysosomal storage diseases.

Like prosaposin, Sap-r contains a number of different, putatively active sphingolipid binding domains. In vertebrates, four of those domains are present, which are cleaved to yield four different saposins with specificity towards different sphingolipids. Prosaposin deficiency in mice or men therefore leads to very severe, lethal storage phenotypes because degradation of most sphingolipid subclasses is affected, as opposed to mutations in specific hydrolases or point mutations in one of the four SapB domains, with storage of one specific subclass.

*Drosophila* Sap-r contains eight putatively active SapB domains, opening up the question of whether the higher number of domains leads to a greater variety in specificity. Crystal structures suggest that the matured vertebrate saposins can form dimers or even multimers, depending on pH and the presence of detergent substances, and it was proposed that lipid binding and solubilization might occur in the hydrophobic cavities formed by multimeric saposin complexes (Ahn et al., 2006). The exact mechanism by which saposins act on lipids remains elusive, which hampers predictions in terms of lipid specificity of the *Drosophila* SapB domains. However, *in silico* analysis revealed that a higher number of SapB domains (typically 8) probably constitutes an autapomorphy of the clade of Arthropoda, which opens up the question of whether this correlates with specifics of lipid composition in arthropods. There are certain differences in sphingolipid composition between *Drosophila* and mammals, such as a shorter sphingoid base alkyl chain with ∼14 C atoms vs ∼18 in mammals, and longer fatty acyl chains attached to it by an amide linkage (Acharya and Acharya, 2005). Furthermore, glycosphingolipids in *Drosophila* contain the core mactosyl-ceramide instead of lactosyl-ceramide in mammals, and PE-ceramide fulfils roles similar to sphingomyelin, which is not present in flies. It remains to be seen if any of these differences account for the increased number of SapB domains in fly Sap-r.

Taken together, our data shows that *Drosophila Sap-r*-null mutants are a suitable model to study lysosomal storage diseases and show all major hallmarks of these diseases, like sphingolipid storage, an enlarged endo-/lysosomal compartment, reduced viability, progressive neurodegeneration, oxidative stress and defective autophagy. Although lysosomal dysfunction was recognized as the cause of LSDs in the middle of the last century (Hers, 1965), it is still a major task to unravel the complex mechanisms involved in LSD pathology. While the primary consequences of lysosomal dysfunction involve the storage and therefore depletion of materials that should be recycled in lysosomes, secondary effects are diverse and can involve altered signaling cascades, increased oxidative stress and changed calcium homeostasis, to name just a few (Ballabio and Gieselmann, 2009). During the review process of this article, another group published results obtained from a different *Drosophila Sap-r* allele (*Sap-r^C27^*; Hindle et al., 2017), which are consistent with our studies. Whereas the *Sap-r^C27^* flies are homozygous viable and show a milder phenotype, probably due to the expression of residual *Sap-r-RB* transcript, our *Sap-r^2.2^* allele is a true null allele and shows a stronger phenotype with respect to the lethality profile and lipid homeostasis. We also generated a *Sap-r* allele (*Sap-r^10.1^*) that still contains the alternative start codon, which could give rise to a truncated protein made from transcript *Sap-r-RB*. These flies also have minimal, almost undetectable levels of RB transcript left, show a much milder phenotype, and are homozygous viable, consistent with the findings of Hindle et al. (2017).

It has recently become apparent that most LSDs lead to defects in autophagic flux as a consequence of lysosomal dysfunction as well as an increase in autophagy induction, especially in sphingolipidoses, and some authors even suggest to understand LSDs as ‘autophagy disorders’ (Lieberman et al., 2012). This aspect of the disease is also present in our *Drosophila* model of a sphingolipidosis, making it a suitable model for future studies on disease progression and defective signaling events.

## MATERIALS AND METHODS

### Fly stocks

The *Sap-r^2-2^* mutant was generated as described by Parks et al. (2004) using the lines d00389 and e01294 from the Exelixis collection, Harvard. Mutant candidates were selected by white eye color and confirmed by genomic PCR and real-time RT-PCR. The stock was kept balanced with TM6B GFP, and homozygous animals were recognized by the absence of GFP fluorescence. Heterozygous animals were generated by crossing *w^1118^* virgins with *Sap-r^2.2^*/TM6B-GFP balanced males and collecting non-GFP progeny.

UAS-Sap-r fly lines were created by cloning the *Sap-r* cDNA into pUAST-attB, and injection into flies with landing sites at 51C and 86F, respectively, was done by BestGene Inc., California. Other fly stocks used were act-Gal4, UAS Atg8a-mCherry, UAS Rab7-GFP, UAS Rab5-GFP and Df(3R) Exel 8194 [Bloomington *Drosophila* Stock Center (BDSC)]. Since the mutant parent strains were made in a *w^1118^* background, *w^1118^* were used as control animals. As a second control, we used wild-type Oregon R flies in some experiments (as indicated). The act-FRT-CD2-FRT-Gal4 clonal driver line (BDSC) to induce rescue clones was genomically recombined with *Sap-r^2.2^* using standard *Drosophila* techniques.

### H_2_O_2_ assay and climbing assays

H_2_O_2_ concentration of larval lysates was measured using the Amplex Red Hydrogen Peroxide/Peroxidase Assay Kit from Molecular Probes following the manufacturer's instructions. For the climbing/negative geotaxis assay, newly emerged virgin flies were collected within 8 h and aged in groups of 7-10 individuals. Climbing assay was performed as described elsewhere (Pendleton et al., 2002).

### Immunofluorescence and use of fluorescent dyes

Peptide antibodies against three *Sap-r*-specific epitopes (designated Sap-rI, Sap-rII and Sap-r1, compare Fig. 1A) were ordered from Pineda Antibody Service, Berlin, Germany. The obtained sera were affinity purified via Protein A-coupled Sepharose and tested on tissue and lysates (control versus mutant) and on Sap-r-overexpressing tissues. Secondary antibodies coupled to Alexa 488, Alexa 543 or Alexa 647 were from Molecular Probes and used at 1:200-1:1000. Filipin staining was done as described elsewhere (Huang et al., 2007). Fly tissue was dissected on ice and fixed in 4% formaldehyde in 1×PBS for 20-40 min or stained with LysoTracker Red/MitoTracker Green FM (both Molecular Probes) before fixation. After MitoTracker Green/LysoTracker Red double staining, as well as single LysoTracker Red staining, live tissue was mounted and imaged immediately. Imaging was done with a Zeiss LSM 710 microscope and images processed using Image J/Fiji and Photoshop software.

### Quantification of Propidium Iodide staining

Five optical lobes per genotype were scanned with identical settings (25× objective, zoom 0.8, optimal resolution in *x/y* and *z*, full use of dynamic range of 8-bit, pinhole 1 AU). Maximum intensity projections of 7 consecutive optical sections were generated for each optical lobe scanned. Analysis was done with Image J/Fiji. The area of the optical lobe was cropped by hand, and threshold was set to 94 of 255. The ‘Despeckle’ function was used to reduce noise. Number of PI-positive particles was then analyzed using ‘Analyze Particles’.

### Histology and TEM

For semi-thin sections, heads of 6- and 30-day-old *w^1118^* and *Sap-r^−^*/Df 8194 female adult *Drosophila melanogaster* were fixed in 4% formaldehyde in 1× PBT (PBS with 0.1% Tween-20) for 4 h at room temperature (RT), dehydrated stepwise in a graded ethanol series and embedded in JB-4 (Polysciences, Inc.). Semi-thin sections were cut at 5 µm with an ultramicrotome with glass knives and stained with Hematoxylin and Eosin.

For transmission electron microscopy (TEM), heads, abdomen and guts of 34-day-old *w^1118^* and *Sap-r^–^*/Df8194 female adult *Drosophila melanogaster* were processed as described in Lehmacher et al. (2012) with minor modifications. Briefly, heads, abdomen and guts were prepared and fixed for 4 h at RT in 2% glutaraldehyde (Sigma, Germany)/4% paraformaldehyde (Merck, Germany) in artificial hemolymph (Lehmacher et al., 2012), subsequently washed in 0.5 M cacodylate buffer pH 7.4, post-fixed for 2 h at RT in 1% osmium tetroxide in 0.5 M cacodylate buffer pH 7.4 (Science Services, Germany), dehydrated stepwise in a graded ethanol series and embedded in Epon 812 (Fluka, Buchs, Switzerland). Ultra-thin sections (70 nm, ultramicrotome EM UC6, Leica, Wetzlar, Germany) were afterwards stained for 30 min with 1% uranyl acetate (sciences services, Germany) and 20 min in 3% lead citrate (Roth, Germany). TEM images were acquired with a Zeiss 902 transmission electron microscope.

### Lethality assays

L1 larvae were collected (*Sap-r* mutants were recognized by the absence of GFP-balancers) on apple juice agar plates (20-25 larvae per plate) and supplied with yeast paste, or on plates filled with JazzMix instant cornmeal food (Fisher Scientific). The number of emerging pupae, pharates and adults (alive 24 h after hatching) was counted.

### Real-time RT-PCR

Whole RNA of dissected adult guts or larval fatbodies was isolated using the Nucleospin RNA kit (Macherey and Nagel). Tissue was homogenized using a Precellys 24 homogenizer (Peqlab). Transcription to cDNA was performed using the Quantitect Reverse Transcription Kit (Qiagen). Quantitative PCR was performed with a CFX Connect cycler (Bio-Rad). Each experiment was repeated at least 5 times.

### Statistics

Two-tailed heteroschedastic Student's *t*-test was applied for normally distributed single comparisons. Kruskal-Wallis/Mann-Whitney *U*-test was used as non-parametric test. The Shapiro-Wilk test was applied to test normality. *P<0.05, **P<0.01, ***P<0.001. All error bars represent standard error of the mean (s.e.m.), except for mass spectrometry data, where they represent standard deviation (s.d.).

### Lipid analysis by TLC

Male flies (6 and 27 days old) were homogenized in water, lyophilized and weighed. This corresponds to the dry weight of the sample; 10 mg of dry weight corresponds to approximately 45 flies. Lipids were sequentially extracted for 24 h at 37°C in each of three solvent mixtures chloroform/methanol/water (2/4/1, v/v/v), chloroform /methanol (1/1, v/v), chloroform/methanol (2/1, v/v) (Reichelt et al., 2004). Cell debris was removed by centrifugation (200 ***g***, 10 min). The united lipid extracts were evaporated in a stream of nitrogen. For the analysis of glycerophospholids, a part of the sample was separated into acidic and neutral lipids by anion exchange chromatography with DEAE-cellulose (Momoi et al., 1976) and lipids were desalted by reversed-phase chromatography on LiChroprep RP18 (Merck, Darmstadt, Germany).

For a better analysis of sphingolipids, another subsample was degraded by mild alkaline hydrolysis with 50 mM sodium hydroxide in chloroform/methanol (1/1, v/v) for 2 h at 37°C. After neutralization with glacial acetic acid, lipids were desalted by reversed-phase chromatography and separated into acidic and neutral lipids by anion exchange chromatography with DEAE-cellulose, followed by desalting by reversed-phase chromatography.

Lipids were applied to prewashed thin layer Silica Gel 60 plates (Merck, Darmstadt, Germany) [chloroform/methanol (1/1, v/v)]. Sphingolipids and phospholipids were separated by TLC with chloroform /methanol/water (60/25/4, v/v/v). Cer and sterol were developed with chloroform /methanol/acetic acid (190/9/1, v/v/v).

For quantitative analytical TLC determination, increasing amounts of standard lipids [CerPE d17:1/12:0, LacCer d18:1/16:0 (all Avanti Polar Lipids), PS 16:0/16:0, PI (from bovine liver), Cer 18:1/18:0, cholesterol, GalCer (from bovine brain, equates glucosylceramide under the chosen conditions; all from Sigma-Aldrich)] were applied.

Plates were air dried, sprayed with 8% (w/v) phosphoric acid containing 10% (w/v) copper (II) sulfate pentahydrate, and charred for 10 min at 180°C. Lipids were quantified by photo densitometry (Camag, Muttenz, Switzerland) at 595 nm.

### Mass spectrometric analysis with a hybrid quadrupole Orbitrap tandem mass spectrometer

#### Chemicals, solvents and lipid standards

Common chemicals and solvents of ACS or LC-MS grade were from Sigma-Aldrich or Fluka (Buchs St Gallen, Switzerland); methanol (LiChrosolv grade) was from Merck (Darmstadt, Germany). Synthetic lipid standards were purchased from Avanti Polar Lipids (Alabaster, AL, USA). 10 µl of lipid standards mixture contained: PE, 12:0/13:0 (50 pmol); PG, 12:0/13:0 (10 pmol); PS, 12:0/13:0 (40 pmol); PC, 12:0/13:0 (40 pmol); PI, 12:0/13:0 (50 pmol); TAG, d5 16:0/18:0/16:0 (20 pmol); Ceramide, d18:1/12:0 (20 pmol); Gal-Ceramide, d18:1/12:0 (20 pmol); PE-Ceramide, d18:1/12:0 (20 pmol).

#### Tissue extraction

Flies were extracted according to the Folch procedure as follows: for each extraction, five flies were added to 500 µl of chloroform/methanol (2/1, v/v) and ∼50-100 µl zirconium beads 1 mm diameter (Biospec Products) in 1.5 ml reaction vials (Eppendorf). Flies were homogenized for 1 min at maximum speed using a TissueLyser (Qiagen GmbH). After this treatment, animals were completely disrupted as observed under a stereomicroscope. 25 µl of this homogenate was spiked with 10 µl of lipid standards mix (see above) and the volume adjusted to 200 µl with chloroform/methanol (2/1, v/v). This homogenate was extracted by adding 40 µl of 150 mM ammonium acetate, pH 8, and shaking for 1 h at 5°C. Extracts were centrifuged for 5 min at 3000 rpm in a MiniSpin centrifuge (Eppendorf) and the lower organic phase was recovered into 2 ml glass vials (Supelco), dried overnight in a vacuum desiccator and resolubilized with 100 µl methanol/chloroform (2/1, v/v).

#### Mass spectrometry

A 10 µl aliquot of sample extract was mixed with 10 µl of either 13 mM ammonium acetate in isopropanol or 0.05% (v/v) triethylamine in methanol in a 96-well plate (Eppendorf, Germany). Samples were infused via the robotic nanoflow ESI source Triversa NanoMate (Advion BioSciences, Ithaca NY) into a hybrid quadrupole Orbitrap tandem mass spectrometer Q Exactive (Thermo Fisher Scientific). The robotic ion source was controlled by Chipsoft 8.3.1 software; backpressure was 0.8 psi and ionization voltage 1.2 kV in negative mode. Ion transfer tube temperature was set to 200°C and S-Lens level was set to 50. Samples were measured in positive and negative ionization mode using a targeted MS2-method (t-MS2) using an inclusion list between m/z 400.5 and 1000.5. The width of the precursor isolation window was set to 1 Th and was centered on each half integer m/z (e.g. 400.5; 401.5; …). Full MS spectra were acquired under the targeted mass resolution *R*=140,000 [full width at half maximum (FWHM) at m/z 200]; target value for the automated gain control (AGC) of 1×106 and maximum ion injection time of 50 ms. MS/MS spectra were acquired with the target mass resolution *R* m/z 200 of 70,000; target AGC value of 1×105; maximum ion injection time of 1000 ms. Normalized collision energy was set to 25%. Run time for each sample was 10 min, during which time, each m/z value of the inclusion list was subjected to MS/MS measurement at least twice.

#### Data processing

Data files were imported into LipidXplorer as described (Herzog et al., 2013). MS and MS/MS spectra were imported with intensity thresholds of 10× the respective signal-to-noise ratios as reported by the Xcalibur software (Thermo Fisher Scientific, Bremen, Germany) and allowing a mass tolerance of 5 ppm. Lipids were identified using the LipidXplorer software (Herzog et al., 2011, 2012, 2013) by matching the the m/z values of the monoisotopic precursors and lipid specific fragments to corresponding elemental composition constraints. Where possible, lipid species were considered identified only if present in positive and negative ionization mode. Lipids were quantified by normalizing the intensities of the identified precursor and fragment peaks of the lipid species to those of their respective internal standards.

#### Data analysis and visualization

Concentrations of lipids determined by mass spectrometry were log transformed and moderated T statistics calculated using R and limma. *P*-values were corrected for multiple testing using the Benjamini and Hochberg method. Lipids with a log2 fold change greater than 1 and smaller than −1 with an fdr<0.01 were selected for unsupervised clustering and heatmap presentation of the data.

Clustering was performed after scaling and centering using euclidean distances and complete linkage as agglomeration algorithm. Rows were split into four clusters by k-means clustering.
